# The Degradation Characteristics and Soil Remediation Capabilities of the Butachlor-Degrading Strain DC-1

**DOI:** 10.3390/microorganisms12122568

**Published:** 2024-12-13

**Authors:** Yue Cheng, Qian Fu, Guixin Xiong, Yaning Huang, Xu Li, Qingyue Yu, Fuxia He, Haitao Li, Rongmei Liu

**Affiliations:** College of Life Sciences, Northeast Agricultural University, Harbin 150030, China; chengyue1523@163.com (Y.C.); fuqian9612@163.com (Q.F.); 18946260939@163.com (G.X.); s220902020@neau.edu.ca (Y.H.); lixu971106@163.com (X.L.); yuqingyue1991@neau.edu.cn (Q.Y.); hefuxia969@163.com (F.H.)

**Keywords:** herbicide degradation, *Bacillus*, bioremediation, liquid chromatography, soil microbial community

## Abstract

Butachlor is a widely utilized acetamide herbicide noted for its systemic selectivity against pre-emergence grass weeds. Butachlor has negative effects on organisms and the environment, so it is necessary to screen degradation strains. In this investigation, *Bacillus cereus* strain DC-1 was isolated from soil persistently exposed to butachlor. Through rigorous single-factor and response surface analyses, strain DC-1 exhibited a notable 87.06% degradation efficiency under optimized conditions where the temperature was 32.89 °C, pH was 7.29, and inoculum concentration was 5.18%. It was further hypothesized by LC-MS that the degradation pathway of butachlor by strain DC-1 might be as follows: butachlor undergoes initial deoxygenation catalyzed by dioxygenases to form 2-chloro-N-(2,6-diethylphenyl)-N-methylacetamide, followed by N-demethylation yielding 2-chloro-N-(2,6-diethylphenyl) acetamide, and culminating in conversion to 2,6-diethylphenol. In addition, bioremediation experiments of butachlor-contaminated soil were conducted. The results show that strain DC-1 could degradable 99.23% of butachlor (100 mg·kg^−1^) from the soil within 12 d, and soil sucrase, cellulase, and urease activities are promoted by the bacteria. And through high-throughput sequencing, it was concluded that the strain DC-1 was able to influence the relative abundance of certain bacteria in the soil, and make the microbial community in the soil develop in a more stable and beneficial direction. DC-1 thus represents a valuable resource in the realm of butachlor degradation due to its robust efficacy, favorable characteristics, and ecological restorative capabilities, underscoring its promising role in the bioremediation of butachlor-contaminated soils.

## 1. Introduction

Butachlor, an acetamide-type systemic herbicide, acts pre-sprouting and is primarily used for the control of annual grass weeds; the half-life of acetochlor in surface soil is 6.51–13.9 days [1,2]. In India alone, the annual usage of butachlor has reached 6.75 × 10^6^ kg [3]. In 2018 and 2019, the concentrations of butachlor in the Babol Roud River in northern Iran were 299.6 and 413.2 μg·L^−1^ [4], respectively. The content of butachlor in the soil of farms in Golestan Province, Iran, reached 0.016 mg·kg^−1^ [5]. At 20 °C, the water solubility of butachlor is 20 mg·L^−1^. It contaminates groundwater and surface water globally through natural diffusion and spray drift mechanisms, accumulating in fish, biota, and terrestrial environments, thereby exerting toxic effects on various organisms exposed to it and impacting public health [4,6]. Butachlor negatively affects organisms such as microcystis aeruginosa and goldfish [7,8], and is acutely toxic to earthworms [9]. Butachlor is toxic to animals which underscores its potential harm to humans, causing direct or indirect damage to the body. Studies indicate that butachlor causes the disruption of lipid metabolism and hepatic damage in mice [10]. Microbiological analyses showed that the abundance and diversity of gut microbiota in mice are significantly reduced by exposure to butachlor, and also dramatically altered the composition of gut microorganisms [11]. It is carcinogenic, generating reactive oxygen species (ROS) upon exposure, which can induce malignant transformation through mechanisms such as stimulating cell proliferation, chromosomal breakage, and DNA damage in vitro. Additionally, it disrupts the endocrine system and potentially triggers immune toxicity reactions internally [1,12,13]. Classified as highly neurotoxic and genotoxic, there are potential biological and ecological threats from butachlor [14,15]. Therefore, the screening of butachlor-degrading bacteria is important.

While various physical and chemical processes such as chemical hydrolysis, photolysis, and adsorption can degrade butachlor in the soil, inexpensive and environmentally friendly bioremediation technologies represent superior methodologies [16]. Currently, numerous microorganisms known for their ability to degrade butachlor have been identified [17,18]. These strains include *Bacillus* sp. Hys which can degrade >90% butachlor concentration within 7 days at a concentration of 100 mg·L^−1^ [19]; *Rhodococcus* sp. T3-1 which can completely degrade 100 mg·L^−1^ butachlor within 6 days [20]; *Bacillus altitudinis* A16 which can degrade 90% of 50 mg·L^−1^ butachlor [16]; *Catellibacterium caeni* sp. nov DCA^−1^T which can degrade 81.2% of 50 mg·L^−1^ butachlor within 84 h [21]; *Pseudomonas putida* G3 which can completely degrade up to 360 mg·L^−1^ butachlor within 700 h [22]; *Ammoniphilus* sp. JF can completely degrade 100 mg·L^−1^ butachlor within 24 h [23]; *Proteiniphilum* sp. BAD-20 which can degrade 90% of mg·L^−1^ butachlor in 10 days [24]; *Paracoccus* sp. FLY-8 which can degrade 85% of 50 mg·L^−1^ butachlor within 5 days [25]. This study selected a strain from butachlor-contaminated soil and was able to degrade more than 80% of butachlor within 12 h at a concentration of 100 mg·L^−1^. The strain was identified as *Bacillus cereus* by 16S rRNA sequencing and physiological indexes, and was designated as DC-1. The degradation capacity of strain DC-1 exceeded that of other degrading bacteria, and it demonstrated excellent biodegradation ability.

There are four main degradation processes for butachlor amine: (i) In the first pathway, butachlor first forms N-(butoxymethyl)-N-(2-chloroethyl)-2,6-diethylaniline by deacylation and the loss of acetyl groups, which successively catalyzes the formation of N-(butoxymethyl)-2,6-diethyl-N-propylaniline and N-(butoxymethyl)-2-ethyl aniline [14,21]. (ii) In the second pathway, dealkylation reactions at different positions resulted in different degradation products and directions of degradation, including C-dealkylation and N-dealkylation. (iii) For the third pathway, the hydrolysis of the C-N of the butyrylamine branch chain and the products are 2-chloro-N-(2,6-diethylphenyl)acetamide and butoxymethanol. In addition, several functional enzymes, such as ChlH, CndA, CndB1, and CndC1, were shown to play a role in the N-dealkylation of butachlor amine, which was degraded to N-chloroacetyl-2,6-diethylaniline and butoxymethanol in the presence of these enzymes [18,26]; for the third pathway, isolated enzyme Dbo was shown to be able to degrade the C=O bond of butyricarbamide, which was degraded by deoxygenase (Dbo) to 2-chloro-N-(2,6-diethylphenyl)-N-methylacetamide, which was then degraded by N-desmethylase to produce N-chloroacetyl-2,6-diethylaniline, which was subsequently transformed into 2,6-diethylaniline [19]. (iv) In the fourth pathway, firstly, the C=O bond of butachlor undergoes hydrolysis to form N-hydroxymethyl-2-chloro-N-(2,6-diethylphenyl)-acetamide. This is then converted to N-chloroacetyl-2,6-diethylaniline by dehydroxymethylation. N-hydroxymethyl-2-chloro-N-(2,6-diethylphenyl)-acetamide is also converted to N-(2,6-diethylphenyl)-N-hydroxymethylacetamide by dechlorination. N-(2,6-diethylphenyl)-N-hydroxymethylacetamide is dehydrogenated by the removal of a methyl group to form N-(2,6-diethyl phenyl)-N-hydroxymethylformamide. Butylcarbamide can also be translated into (2,6-diethylphenyl)-ethoxymethylcarbamic acid by de-ethylation and dechlorination [21].

Soil enzymes act as important indicators of soil fertility and are the driving force of soil metabolism, participating in various biochemical processes in soil and playing crucial roles in carbon balance and phosphorus or nitrogen cycling in the soil environments [27,28]. As integral participants in various biochemical reactions within the soil, soil microbes typically function as complex communities engaged in essential soil life processes. Changes in community structure are sensitive to changes in ecosystem dynamics and soil quality [29,30,31]. There are relevant reports indicating that environmental pollution alters the composition of the soil microbial communities by affecting their metabolic activities [32]. Therefore, the addition of butachlor to soil leads to changes in the structure of the soil microbial community, and soil microbial diversity is affected. Butachlor was effectively removed in the constructed single-compartment microbial fuel cell (MFC), and a significant increase in the number of *Paracoccidioides* and *Graminobacteria* was observed with butachlor as the sole carbon source, which is an important potential use for wastewater bioremediation, sediment, and soil [33]. Studies have shown that the abundance of *Bradyrhizobium*, *Mycobacterium*, and *Anaeromyxobacter* was higher in PAH-contaminated soils under PAH stress [34]. There are reports that butachlor had an effect on different microbial populations in rice soils, different microbial enzyme activities, and microbial substance transformation [35]. In addition, several studies have found that exogenous bacteria and native microorganisms exhibit synergistic effects to promote the growth of petroleum-degrading bacteria in soil and affect soil microbial community composition [32,36]. Furthermore, degrading bacteria led to the remodeling of soil microbial community structure and accelerated the degradation of pollutants in contaminated soil [28]. In conclusion, the ability of degrading bacteria to remediate butachlor-contaminated soils and their impact on soil ecology can be studied by observing changes in the microbial community structure.

This study isolated bacterial strains able to degrade butachlor from soil persistently contaminated with this herbicide. It extensively investigated the degradation characteristics of the strain, identified its degradation genes and pathways, further examined its remediation capacity in butachlor-contaminated soil, and elucidated the impact on the soil enzymes and microbial communities of this strain during the soil remediation process.

## 2. Materials and Methods

### 2.1. Pharmaceuticals and Culture Media

Butachlor reagent (99.6% purity) was purchased from Beijing Qincheng Yixin Technology Co., Ltd. (Beijing, China), and a soil enzyme assay kit was purchased from Suzhou Keming Biotechnology Co., Ltd. (Suzhou, China). Chromatographic grade methanol and acetonitrile were used for high-performance liquid chromatography, and other reagents (e.g., dichloromethane) were analytical grades.

LB medium, pH = 7, containing 10 g of Tryptone, 10 g of NaCl, and 5 g of Yeast extract, was dissolved in 800 mL of distilled water, adjusted to pH 7.0, and filled to 1 L with distilled water.

MSM, adjusted to pH 7.0, comprised the following components per liter: NaCl 1.0 g, NH_4_NO_3_ 1.0 g, K_2_HPO_4_ 1.5 g, KH_2_PO_4_ 0.5 g, MgSO_4_·7H_2_O 0.2 g, and 1 mL of trace elements solution. The solution was prepared by dissolving these salts in distilled water and adjusting the pH to 7.0, and then diluting to a final volume of 1 L.

### 2.2. Enrichment and Isolation of Bacteria

A total of 5 g of the soil sample contaminated with butachlor was inoculated into 100 mL Minimal Salt Medium (MSM), the initial butachlor concentration was set at 50 mg·L^−1^, and stirred at 150 rpm at 30 °C for 7 days under aerobic conditions. Subsequently, 5% (*v*/*v*) of the enriched culture was transferred to a fresh MSM, now containing 100 mg·L^−1^ of butachlor. The process was repeated four times, and the concentration of butachlor was gradually increased to 200 mg·L^−1^. Finally, the enriched cultures from conical bottles were diluted, spread on LB plates, and incubated at 30 °C. Colonies with distinct morphologies were selected and streaked onto new LB plates for the isolation and purification of different strains [37].

### 2.3. Identification of Microbial Strains

Following the guidelines from “Bergey’s Manual of Systematic Bacteriology”, microbial strains were preliminarily identified by observing colony morphology and conducting physiological and biochemical tests. Physiological tests were performed using standard identification tubes. Genomic DNA extracted from strains was used as a template for 16S rRNA gene PCR amplification using universal bacterial primers 27F (5′-AGAGTTTGATCCTGGCTCAG-3′) as the forward primer and 1492R (5′-TACCTTGTTACGACTT-3′) as the reverse primer [38]. The PCR reaction conditions included an initial denaturation at 95 °C for 5 min, followed by 29 cycles of denaturation at 95 °C for 1 min, annealing at 54 °C for 30 s, extension at 72 °C for 90 s, and a final extension at 72 °C for 5 min. The amplified products were analyzed by electrophoresis on a 1% agarose gel and purified by the AxyPrep DNA Gel extraction kit (Shanghai, China). Purified DNA was sequenced by Kumei Biological Company in Jilin Province (Changchun, China). The sequencing results were uploaded to GenBank and obtained the accession number SUB14664896, and the sequences of the strains were compared by the Blast program in the NCBI nucleotide database (https://blast.ncbi.nlm.nih.gov/Blast.cgi (accessed on 14 August 2024), and a phylogenetic tree was constructed using MEGA-X 7.0.26 based on the aligned sequences of closely related species.

### 2.4. Study on the Growth and Butachlor Degradation Capability of Bacterial Strains

In this study, the investigation focused on the degradation and growth conditions of bacterial strains using MSM containing 100 mg·L^−1^ of butachlor. Butachlor served as the carbon source, while ammonium nitrate (1 g·L^−1^) served as the nitrogen source in a 100 mL culture volume. The bacterial suspension (1 × 10^8^ cfu·mL^−1^) was inoculated at 1% (*v/v*) into the MSM and cultured under aerobic, dark conditions at 30 °C with agitation at 150 rpm·min^−1^. During cultivation, OD_600_ was measured every 2 h, and the concentration of butachlor was determined using HPLC. Each experiment was conducted in triplicate to monitor both bacterial growth and butachlor degradation. The effects of various environmental factors of bacteria growth and butachlor degradation were explored through single-factor experimental designs, including pH (4.0, 5.0, 6.0, 7.0, 8.0, 9.0, 10.0, 11.0, and 12.0), temperature (15, 20, 25, 30, 35, 40, and 45 °C), initial substrate concentration (50, 100, 200, 300, 400, 500, and 600 mg·L^−1^), and inoculum size (1%, 3%, 5%, 7%, and 9% *v/v* of bacterial suspension at 1 × 10^8^ cfu·mL^−1^). We used the response surface method to optimize the degradation conditions of butachlor, identifying the optimal parameters based on the butachlor degradation rate as the response variable. Based on the single factor experimental results, the Box–Behnken design (BBD) model was established by te Design-Expert 10 software with the inoculated amount (%), temperature (°C), and pH as the independent variables, and the optimal conditions for butachlor degradation were determined.

### 2.5. Degradation Product Analysis

Extraction of butachlor Degradation Products: The cultured samples were centrifuged at 12,000× *g* for 20 min at room temperature. The supernatant was freeze-dried, dissolved in 1 mL of chromatography-grade acetonitrile, filtered through a 0.22 μm filter, and stored in amber vials for analysis.

Detection: LC-MS analysis of reaction products—The analysis was performed using a time-of-flight mass spectrometer in electrospray ionization (ESI) under positive mode conditions. The flow rate of the conical gas was 50 L·h^−1^, and the flow rate of the dissolved gas was 1000 L·h^−1^, and argon was used as the collision gas and nitrogen as the de-solubilized gas. The chromatography was performed on the Eclipse-XDB-C18 column (150 mm × 4.6 mm, 5 μm) with mobile phase acetonitrile—water = 80:20 (*v/v*). The compound was ionized in an ESI source and detected in high-resolution mode, recording a full-scan signal of m·z^−1^ 50 to 600 [19].

### 2.6. Soil Remediation

The inoculation of strain DC-1 in MSM containing butachlor for enrichment cultivation: Upon reaching an OD_600_ of 1.0, bacterial cells were harvested and washed with sterile water three times [19]. The cells were then resuspended in sterile water to a bacterial suspension concentration of approximately 1 × 10^8^ cfu·mL^−1^. The experimental treatments were as follows: (A) sterilized soil (250 g) + sterile water (8 mL); (B) sterilized soil + DC-1 bacterial suspension (8 mL, 1 × 10^8^ cfu·L^−1^); (C) fresh soil (250 g) + sterile water (8 mL); (D) fresh soil + DC-1 bacterial suspension (8 mL, 1 × 10^8^ cfu·L^−1^). Both sterile water and sterile soil were treated with a high-pressure sterilization method. This was repeated three times for each treatment group. The experiment was conducted at room temperature with daily watering to maintain soil moisture at approximately 50%. Soil samples (10 g each) were collected every two days until day 16 of treatment to measure the residual butachlor content. This experiment aimed to evaluate the remediation effectiveness of strain DC-1 on soil contaminated by butachlor.

Experimental Design: Three treatments were established: (1) the control group (CK) did not spray butachlor and did not inoculate strain DC-1 in potted soil; (2) treatment group (DC) evenly sprayed butachlor solution in potting soil until the final concentration was 50 mg·kg^−1^; (3) treatment group (DCJ) evenly sprayed butachlor solution in potting soil until the final concentration was 50 mg·kg^−1^, then added DC-1 bacterial suspension (1 × 10^8^ cfu·mL^−1^) and mixed thoroughly. Each pot was irrigated with water to ensure consistent soil moisture across treatment groups, with three replicates per treatment. Soil samples were collected using random sampling methods on days 7, 14, 21, and 28 post-application. This was divided into two samples for each soil sample: one for analyzing soil enzyme activities and the other for assessing changes in microbial community structure.

### 2.7. Soil Microbial Analysis

We extracted DNA from the soil samples and performed qualitative and quantitative analysis on the extracted DNA to detect its quality. We used primers 347F (5′-CCTACGGRRBGCAS-CAGKVRVGAAT-3′) and 802R (5′-GACTACNVGGGTWTCTACC-3′) to amplify the V3-V4 variable region of the soil microbial genome 16S rDNA [39]. Afterward, we performed nucleic acid gel validation and purified the product using the AxyPrep DNA Gel Extraction Kit (Shanghai, China). We analyzed the purity of the purified product and quantified it. Using paired-end sequencing technology (Illumina Novaseq 6000 sequencing platform) (Beijing, China), gene sequencing was performed on the small fragment library, and after base calling analysis, it was transformed into the original sequencing sequence. To obtain high-quality sequences, it is necessary to perform quality control on the previously obtained raw sequencing sequences; quality control mainly includes the following processes: firstly, quality filtering, which requires the use of the Trimomatic v0.33 software to perform quality filtering on the raw read sequences obtained from sequencing, filtering out low-quality sequences; then, to obtain the Clean Read sequences with cleared primers, the cutadapt 1.9.1 software was used to identify the primers in the sequence and remove them; next is length filtering, which requires the overlap function in the Usearch v10 software. First, we concatenated the Clean Read sequences of each sample at both ends to obtain a concatenated sequence (of different lengths), and then set different length ranges for length filtering based on different regions; finally, it is necessary to remove the chimeric sequence from the sequence and denoise it. The data2 [40] method in the QIIME2 2020.6 [41] software is a very convenient and effective way to perform this operation; after the above series of quality control processes, the final valid data (Non-timed Reads) were obtained. We divided OTUs/ASVs and obtained species classification for each group based on the sequence composition of OTUs/ASVs. A Principal Component Analysis (PCA) was used to describe the differences between the samples, and CANOCO 5.0 was used for redundancy analysis to evaluate the relationship between environmental factors and dominant bacterial species in the bacterial communities. A Double Matrix Correlation Analysis was used to explore the relationship between soil dominant species, soil enzymes, and bacterial growth. A One-way ANOVA was used to analyze significant differences between the processed samples, with *p* < 0.05 indicating statistical significance. GraphGad Prism8.0 (GraphPad Software, San Diego, CA, USA) was used for statistical calculations and plotting.

## 3. Results and Discussion

### 3.1. Isolation and Identification of Strain DC-1

**Figure 1:**
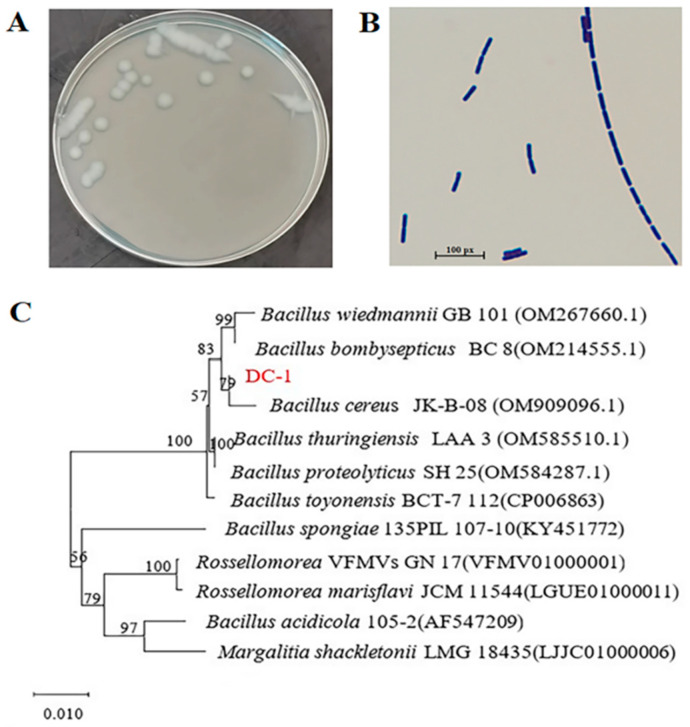
Colony of the strain DC-1 (**A**) and gram staining results (**B**). Phylogenetic tree constructed based on strain DC-1 (marked in red) 16S rRNA sequences (**C**).

Isolation of a strain DC-1 from soil persistently contaminated with butachlor: The colony characteristics of strain DC-1 included large, rough-surfaced, flat, round or nearly round, soft-textured, colorless, slightly glossy white colonies (Figure 1A). Gram staining confirmed the strain as Gram-positive (Figure 1B), and we evaluated its physiological and biochemical properties (Table 1). The size of the 16S rRNA gene sequence was determined to be 1366 bp. Subsequently, the 16S rRNA sequence of strain DC-1 was analyzed and aligned with nucleotide sequences in the NCBI database. Based on the alignment results, a phylogenetic tree of strain DC-1 was constructed using the Neighbor-Joining method in the MEGA-X software. The phylogenetic analysis (Figure 1C) revealed that strain DC-1 clustered closely with *Bacillus cereus*. Combining these findings with morphological characteristics and physiological biochemical analysis, strain DCh-1 was identified as *Bacillus cereus*. *Bacillus* is widely present in ecosystems and plays a significant role in pesticide degradation. *Bacillus* sp. Hys can degrade >90% of butachlor concentration within 7 days at a concentration of 100 mg·L^−1^ and grow on butachlor as the sole carbon source [19]. *Bacillus altitudinis* A16 can degrade 90% of 50 mg·L^−1^ butachlor [16]. *Bacillus cereus* JUN7 can degrade glyphosate [42]. *Bacillus genus* GZT has the ability to simultaneously mineralize 2,4,6-tribromophenol (TBP) and debrominate [43]. These studies indicate that *Bacillus* plays an important role in the degradation of pesticides and has great potential in the bioremediation of environmental pollution. In this study, a strain of bacteria DC-I was selected to degrade butachlor, enriching the bacterial resources for butachlor degradation.

### 3.2. Effects of Environmental Factors on Growth and Biodegradation

**Figure 2:**
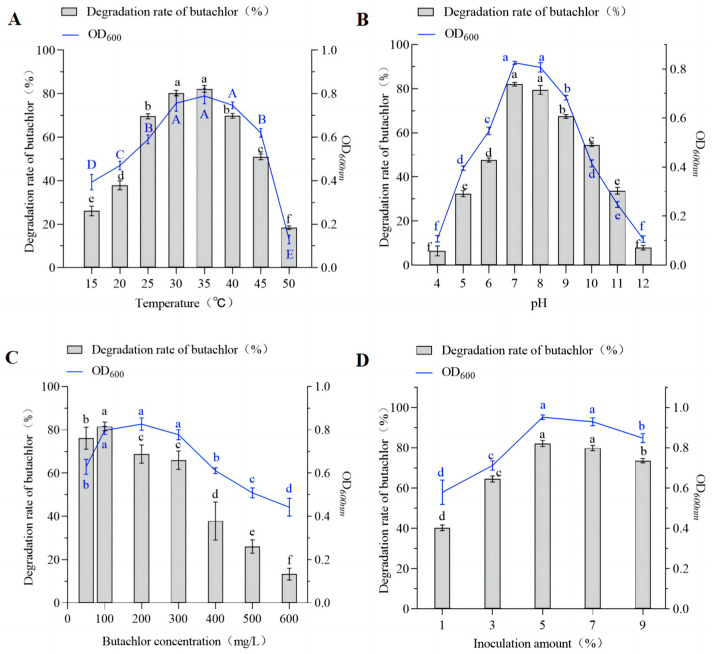
Different temperatures (**A**), pH (**B**), inoculation amount (**C**), and initial concentration of butachlor (**D**). The error bars represent the standard deviation of three replicates. Different letters represent the degree of difference among the different treatments.

Strain DC-1 was grown for 12h at different pH, temperature, substrate concentration, and inoculum amount. We found that strain DC-1 was able to grow and degrade butachlor at temperatures between 30 °C and 40 °C (Figure 2A). Specifically, at 35 °C, strain DC-1 achieved a degradation rate of 82.19% towards butachlor (100 mg·L^−1^) within 12 h, corresponding to maximum growth with an OD_600_ of 0.79. At 15 °C and 50 °C, degradation rates were 26.10% and 28.41%, respectively, although the degradation rate was significantly reduced, the growth of the strain remained good and it was also able to degrade butachlor, proving that the activity of the strain was not completely inhibited. So far, it has been found that the optimum growth temperature of *Proteiniphilum* sp. BAD-20 is 30–35 °C, and this strain shows a significant decrease in the degradation rate of butachlor at temperatures above 35 °C or below 20 °C [24]. *Paracoccus* sp. FLY-8 grows in a temperature range of 15–35 °C [25]. Nevertheless, strain DC-1 was not temperature sensitive and could grow at 5–45 °C. In this temperature range, strain DC-1 still showed a high degradation rate of butachlor. These results suggest that strain DC-1 exhibits adaptability to both lower and higher temperature ranges, maintaining its activity in degrading butachlor.

With increasing pH levels in the culture medium, both the growth of strain DC-1 and its degradation efficiency towards butachlor exhibited a trend of initially increasing followed by decreasing (Figure 2B). At pH 7.0 after 12 h of cultivation, strain DC-1 achieved a butachlor degradation rate of 81.97%, corresponding to maximum growth and degradation efficiency. Subsequently, as pH increased, the degradation of butachlor by strain DC-1, as well as its own growth, gradually decreased. At pH 5.0 and pH 11.0, the degradation rates of butachlor were 32.38% and 33.74%, respectively. As reported in previous studies, strains *Bacillus mojavensis* AZFS18 and *Bacillus paramycoides* AZFS15 showed that they grew fastest at pH = 7.0. When pH < 6.0 or pH > 7.0, both bacteria could hardly grow and chlorantraniliprole (CAP) was hardly degraded [44]. Cycon et al. revealed that the activities of the bacteria and enzymes associated with pesticide transformation may be inhibited at lower pH [45]. Thus, strain DC-1 demonstrates tolerance to a wide pH range, showing higher degradation rates towards butachlor in neutral to slightly alkaline conditions compared to acidic environments.

With increasing inoculum size of strain DC-1, the degradation rate of butachlor also increased after 12 h of cultivation. At an inoculum size of 5%, the degradation rate of butachlor and the growth of strain DC-1 were both maximized, with a degradation rate of 82.10% and OD_600_ of 0.95 (Figure 2C). However, at inoculum sizes of 7% and 9%, both the degradation rate of butachlor and OD_600_ decreased. Adequate nutrients and sufficient space are essential for the optimal growth of bacterial cells, and increased inoculum size may lead to competition among bacterial cells for limited nutrients, thereby restricting their growth. A related report showed that when strain *Sphingomonas chloroacetimidivorans* sp. nov. was inoculated with 5% and incubated for seven days, the results showed that the degradation rate was 68.5 ± 8.7% [46]. In contrast, strain DC-1 had better butachlor degradation ability.

The degradation rate of butachlor reached a maximum value of 81.53% at a concentration of 100 mg·L^−1^. However, as the concentration of butachlor increased, both the degradation rate and OD_600_ began to decline, indicating a potential toxicity effect of high concentrations of butachlor on strain DC-1, thereby affecting its growth (Figure 2D). When the concentration of butachlor was 50 mg·L^−1^, the degradation rate (76.07%) and OD_600_ (0.63) were lower compared to the degradation rate (81.53%) and OD6_00_ (0.80) observed at 100 mg·L^−1^, possibly due to the low concentrations of butachlor providing an insufficient carbon source, which in turn partially inhibited strain growth. Hence, the optimum concentration for the degradation of butachlor by strain DC-1 was 100 mg·L^−1^. In previous studies, *Proteiniphilum* sp. BAD-20 was able to degrade butachlor at an effective concentration of 50 mg·L^−1^; when the concentration of butachlor is increased to 100 mg·L^−1^, butachlor is essentially undegraded [24]. *Pseudomonas putida* strain G3 can tolerate up to 1000 mg·L^−1^ of butachlor, but enters a lag phase with increasing butachlor concentration [22]. The highest concentration of butachlor that strain DC-1 can tolerate is 600 mg·L^−1^.

### 3.3. Optimization of Butachlor Degradation Conditions by Strain DC-1

Effect of environmental factors on the degradation of butachlor by strain DC-1: Optimization through three-factor, three-level response surface methodology is influenced by different environmental conditions, and the effectiveness of strain DC-1 in degrading butachlor was explored using temperature (A), pH (B), and inoculum size (C) as the independent variables in a single-factor experimental design. The degradation rate of butachlor was chosen as the response variable. A response surface analysis was employed to optimize these conditions. A total of 17 groups of experimental plans were obtained using the Box–Benhnken test in Design Expert 10, and 17 groups of experimental results were obtained based on the experimental plans (Table S1). Further regression analysis was conducted on the experimental data to obtain the quadratic equation for the degradation of butachlor by strain DC-1: degradation rate of butachlor (%) = 80.26 + 9.97A + 5.30B + 2.30C + 1.37AB + 2.62AC − 2.78BC − 26.44A^2^ − 28.19B^2^ − 28.04C^2^.

The regression model of the response surface analysis indicated an extremely significant result with *p* < 0.0001, demonstrating its statistical significance. The model’s F-value of 4.49 (F > 0.05) suggests that the lack-of-fit term is not significant, affirming a good fit of the model to the experimental data and high confidence in its predictive capability. The coefficient of determination R^2^ = 0.9928, meaning that the model can explain 99.28% of the response value, and the corrected coefficient of determination R^2^Adj = 0.9836, which is basically close to the value of R^2^, indicating a robust fit and strong correlation of the model. Therefore, this model is suitable for optimizing the environmental conditions affecting butachlor degradation by strain DC-1. Additionally, Table S2 shows that individual factors significantly influence butachlor degradation (*p* < 0.05), while the interactions AB (temperature and pH), AC (temperature and inoculum size), and BC (pH and inoculum size) do not significantly affect butachlor degradation. The interactions are ranked in the following order of significance: BC > AC > AB (Table S2).

Response surface plots provide a clear and intuitive visualization of the interaction between two individual variables. Therefore, using the Design-Expert 10 software, response surface plots were generated to observe the effects of pairwise interactions between inoculation amount, pH, and temperature on the degradation rate of butachlor (response value) predicted by the regression model. On the basis of the analysis of these response surface plots, suitable conditions for butachlor degradation by strain DC-1 were determined to be temperature of 32.89 °C, pH of 7.29, and inoculum size of 5.18% (Figure S1A–C). It has been reported that temperature, pH, and inoculation amount can affect the degradation efficiency of Pseudomonas sp. ZXY and *Pseudomonas putida* strain G3. According to response surface analysis, *Pseudomonas* sp. ZXY showed the highest degradation efficiency at 30.71 °C, pH = 7.14, and 4.23% (*v/v*) inoculation, while *Pseudomonas putida* strain G3 showed the highest degradation efficiency at 32.5 °C, pH = 7.5, and 10% (*v/v*) inoculation [22,47]. Therefore, the optimal conditions for pesticide degradation by different strains are related to the influence and tolerance range of the strains themselves. Under these conditions, the model predicted that strain DC-1 would achieve a butachlor degradation rate of 81.53% within 12 h. The cultivation of strain DC-1 under the optimized conditions resulted in an actual butachlor degradation rate of 82.68% within 12 h, which closely matched the predicted value. This indicates a good fit between the predicted and actual values, validating the effectiveness of the response surface methodology in optimizing the cultivation conditions for strain DC-1.

According to the consequence of single-factor experiments and response surface design, the degradation ability of strain DC-1 towards butachlor was re-evaluated before and after optimizing cultivation conditions (Figure S1D). Following optimization (temperature 32.90 °C, pH 7.29, and inoculum size 5.18%), the degradation rate of butachlor significantly increased compared to the pre-optimization conditions (pH 7.0, inoculum size 5%, and temperature 30 °C). Specifically, prior to optimization, strain DC-1 exhibited a degradation rate of 81.07%, whereas after optimization, this rate increased to 87.06%, marking a 6.65% improvement in degradation efficiency. Moreover, post-optimization, the growth rate of strain DC-1 accelerated, reaching its maximum at 8 h. The increased number of strains resulted in a greater utilization of butachlor, thereby enhancing its degradation efficiency.

### 3.4. Analysis of Degradation Pathways by Strain DC-1

**Figure 3:**
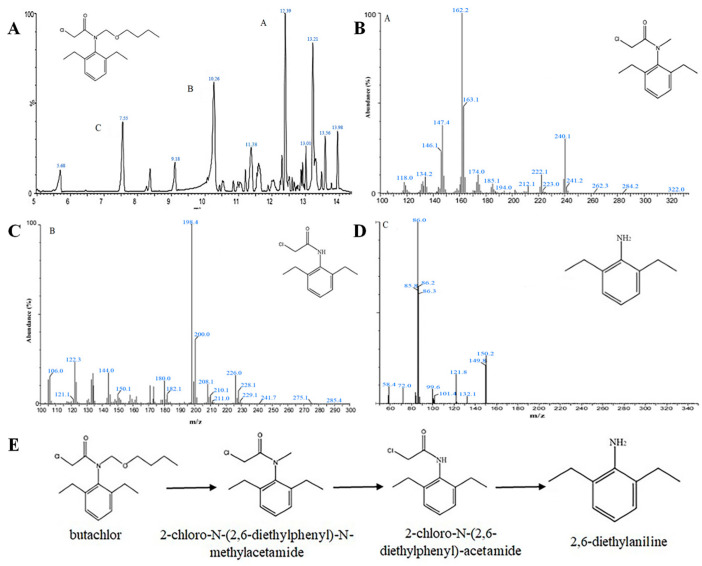
LC-MS detects intermediate metabolites during the degradation of butachlor by strain DC-1: butachlor (**A**), 2-chloro-N-(2,6-diethylphenyl)-N-methylacetamide (**B**), 2-chloro-N-(2,6-diethylphenyl)-acetamide (**C**), and 2,6-diethylaniline (**D**). Degradation pathway of butachlor by strain DC-1 (**E**).

Studies on the degradation pathways of butachlor have focused on the degradation of butachlor by various bacteria [16,19,21]. To investigate the biometabolic process of butachlor degradation by strain DC-1, the intermediates of strain DC-1 butachlor degradation were identified using LC-MS, and three intermediate metabolites with varying retention durations were discovered: metabolites A, B, and C. The intermediate metabolites were also identified by LC-MS and compared to reference mass spectra from the National Institute of Standards and Technology in the United States (Figure 3A). The metabolites exhibited a protonated molecular ion peak [M+H]+ at m·z^−1^ 240.1, as well as fragment ion peaks at m·z^−1^ 174.0, 162.2, 147.4, and 134.2. Based on these findings, product A was identified as 2-chloro-N-(2,6-diethylphenyl)-N-methylacetamide (Figure 3B). When metabolite B was positively ion chemically ionized, a quasimolecular ion peak [M+H]+ of m·z^−1^ 226.0 was seen, along with distinctive fragment ion peaks of m·z^−1^ 208.1, 198.4, 180.0, and 122.3 (Figure 3C). Based on these numerical characteristics, product B was identified as 2-chloro-N-(2,6-diethylphenyl)acetamide. After 6 h of incubation, the concentration of metabolite C rose, and the mass spectrum revealed a base peak at m·z^−1^ 150.2 [M+H]+ with typical fragment ion peaks at m·z^−1^ 132.1, 121.8, and 105.1 (Figure 3D). 2,6 diethylaniline was determined to be the product C based on these characteristics.

In the control group without the inoculation of strain DC-1, the LC-MS analysis conducted at the end of cultivation did not detect peaks corresponding to the three aforementioned metabolites (A, B, and C). This absence demonstrates that strain DC-1’s butachlor breakdown was the particular cause of the production of metabolites A, B, and C.

Based on the above results, the LC-MS analysis of the metabolites generated by strain DC-1 during butachlor degradation revealed the production of three new compounds: 2-chloro-N-(2,6-diethylphenyl)-N-methylethanamide, N-chloroethyl-2,6-diethylphenylamine, and 2,6-diethylphenylamine. These findings suggest a preliminary pathway for butachlor degradation by strain DC-1: initially, butachlor undergoes dealkylation to form 2-chloro-N-(2,6-diethylphenyl)-N-methylethanamide, which then undergoes N-demethylation to transform into N-chloroethyl-2,6-diethylphenylamine. Subsequently, it is further converted to 2,6-diethylphenylamine (Figure 3E). This is consistent with the pathway by which strain *Bacillus* sp. hys-1 degrades butachlor [19]. Strain DC-1 may act synergistically with certain other microorganisms in the environment to eventually completely mineralize the degradation product 2,6-diethylaniline, thus completely eliminating the pollution of the soil environment by butachlor, an aspect that will continue to be thoroughly investigated in subsequent experiments.

### 3.5. Bioremediation of Butachlor-Contaminated Soil

In an inorganic salt medium containing butachlor (100 mg·L^−1^), strain DC-1 exhibited significant degradation capability, with over 80% of the butachlor degraded within 12 h (Figure S1D). It was found that inoculation with DCA^−1^T accelerated the rate of degradation of butachlor in a non-sterile environment and within 5 days, it can degrade 90.4% of 50mg·kg^−1^ butachlor [21], and *Bacillus altitudinis* strain A16 completely degraded butachlor (50 mg·L^−1^) in soil within 5 d [16]. Strain *Rhodococcus* sp. AC-1 was able to completely degrade acetochlor at an initial concentration of 8.4 mg·kg^−1^ in 7 d [48]. This study studied the remediation impact of strain DC-1 on butachlor-contaminated soil, as well as the strain’s capacity to break down butachlor in soil. The results showed that after the addition of strain DC-1, the degradation rate of butachlor (100 mg·kg^−1^) in treatment groups B and D reached 98.9% and 99.23% within 12 d, respectively, and in treatment groups A and C, the degradation rate of butachlor (100 mg·kg^−1^) was only 12.59% and 21.70% at 14 d. Therefore, strain DC-1 significantly improved the soil butachlor degradation efficiency (Figure S2). Compared with treatment groups B and D, the degradation efficiency of butachlor in treatment group D was higher than that in treatment group B. Compared with treatment groups A and C, the degradation efficiency of butachlor in treatment group C was higher than that in treatment group A. This may be owing to the presence of particular bacteria in the soil, which enhances the breakdown of butachlor. Furthermore, the degradation rate of butachlor in treatment group A reached 14.57% after 16 d of incubation, which may be due to the hydrolysis of butachlor [49]. Strain DC-1 had a strong degradation effect in soil and was capable of degrading larger concentrations of butachlor; hence, strain DC-1 might be a promising choice for butachlor-contaminated soil remediation.

### 3.6. The Impact of Butachlor on Soil Enzymes

Soil enzymes are commonly employed as essential markers of soil quality and biological activity [50]. Soil urease is a key enzyme for nitrogen transformation, and its activity evaluates soil microorganisms’ capacity to catabolize and metabolize herbicides as a source of nitrogen [51]. Soil sucrase, cellulase, and catalase are linked to defense mechanisms and the cycling of carbon [10,52]. Therefore, these four enzymes were selected to explore the effect of butachlor on soil enzyme activities.

**Figure 4:**
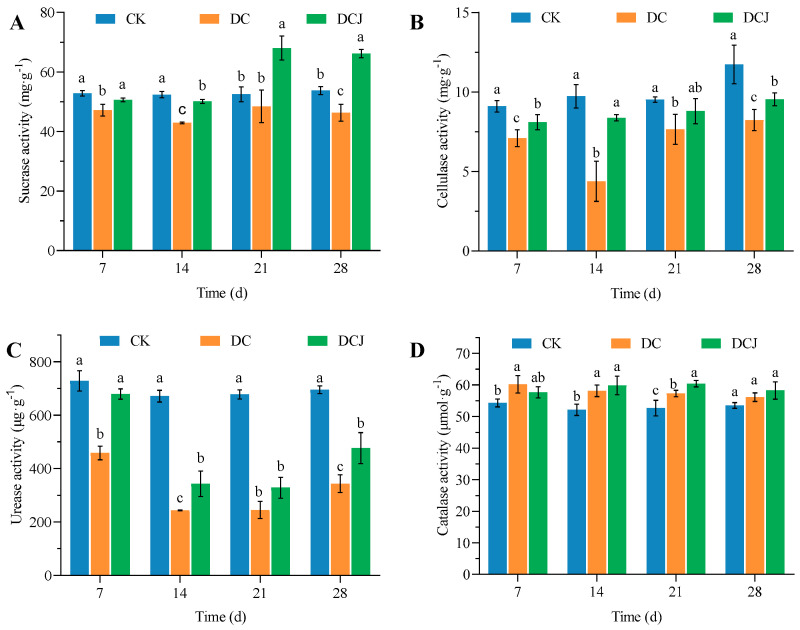
The effect of strain DC-1 on soil enzyme activity treated with butachlor: sucrase (**A**), cellulase (**B**), urease (**C**), and catalase (**D**) (*p* < 0.05; “a, b, c”: It is not significant when it is the same letter, but significant when it is different letters.). Note: CK: without butachlor and strain DC-1; DC: with added butachlor (50 mg·kg^−1^); DCJ: with butachlor (50 mg·kg^−1^) and strain DC-1.

Butachlor can inhibit the activity of soil sucrase enzymes. Seven days after adding butachlor, the inhibition of sucrase in the soil is most pronounced, with enzyme activity reduced to 42.89 mg·g^−1^, an 18.31% decrease compared to the control (CK) (Figure 4A). As butachlor degrades, soil sucrase activity gradually recovers. Strain DC-1 alleviates butachlor’s inhibition of soil sucrase. After inoculating with strain DC-1 for 7 days, soil sucrase activity increases to 50.64 mg·g^−1^, which is 107.33% of the treatment group DC’s activity (Figure 4A). At 21 and 28 days, enzyme activity in the treatment group DCJ notably improves, surpassing that of the CK. Butachlor exhibits inhibitory effects on soil enzyme activity, but strain DC-1 facilitates the restoration of sucrase activity in butachlor-contaminated soil. Over time, the soil sucrase activity continues to recover and even exceeds that of the CK (Figure 4A). This is in agreement with the findings of [53], which showed that 10 mg·kg^−1^ butachlor treatment showed significant inhibition of sucrase activity in the soil, and 2 mg·kg^−1^ and 4 mg·kg^−1^ of butachlor had very little effect on sucrase activity throughout the experimental period and did not show any significant difference [54].

Butachlor inhibits the activity of cellulase enzymes in the soil, which is restored by strain DC-1. Following the addition of butachlor, cellulase activity in soil begins to decline, reaching its lowest point after 14 days of incubation at 4.38 mg·g^−1^, with only 45.02% of the enzyme activity observed in the CK (Figure 4B). As butachlor degrades in soil, cellulase activity gradually recovers in DC and DCJ, with the latter consistently exhibiting higher activity than DC, indicating that strain DC-1 mitigates the pressure of butachlor on cellulase enzymes and promotes their recovery (Figure 4B). In a similar vein, researchers studied the effect of acetamiprid on earthworm cellulase and discovered that acetamiprid inhibited cellulase activity, and the greater the concentration of acetamiprid, the stronger the inhibition [55].

Butachlor suppresses soil urease activity, which is enhanced by strain DC-1 in butachlor-contaminated soil. Following a 7-day butachlor administration, the DC’s urease activity dramatically dropped by 37.10% in comparison to the CK (Figure 4C). Concurrently, the DCJ exhibited markedly higher urease activity at 679.16 μg·g^−1^ (Figure 4C). As butachlor degraded in soil over time, both DC and DCJ treatments showed a decline in urease activity, which partially recovered by day 28 (Figure 4C). During the incubation period, urease activity in both DC and DCJ remained lower than in the CK, but consistently greater in the DCJ compared to the DC, demonstrating that strain DC-1 mitigates the inhibitory impact of butachlor on soil urease activity to some extent (Figure 4C). Wang et al. showed that butachlor inhibits soil urease activity, but urease activity improves in the later stages of incubation, the same conclusion as in this investigation [56]. However, it was also shown that at very low concentrations of butachlor, it had a stimulating effect on urease on the 1st and 7th d, and then inhibited urease activity, and at very high concentrations of butachlor, it showed a continuous inhibition of urease activity, and inoculation with bacteria could increase urease activity in contaminated soils [57].

In contrast to its effect on other enzymes, butachlor activates soil catalase activity. In the CK, soil catalase activity measured 54.28 μmol·g^−1^ (Figure 4D). Both DC and DCJ treatments exhibited higher catalase activity than CK, peaking at 59.12 μmol·g^−1^ and 59.81 μmol·g^−1^, respectively, after 14 days (Figure 4D). This represents an increase of 11.87% and 12.89% compared to CK (Figure 4D). As butachlor degrades, the stimulatory effect of DC and DCJ treatments on catalase activity diminishes, though their activity remains higher than that of the CK (Figure 4D). Therefore, butachlor promotes catalase activity, and throughout the incubation period, the application of strain DC-1 maintains or even enhances butachlor’s activation of soil catalase activity.

Soil enzyme activities can represent soil health for analyzing the effects of pesticides on the soil ecology [58]. Inoculation with strain DC-1 may boost the activities of cellulase, sucrase, and urease in butachlor-contaminated soil while decreasing butachlor’s inhibition of soil enzymes [41]. This was also mentioned in the study of soil ecological remediation of atrazine by degrading bacteria; strain XMJ-Z01 was an effective way to remediate simazine-contaminated soil [59]. Exogenous bacteria increase the activity of important enzymes in the soil [60], promoting the proliferation of soil microorganisms and accelerating the decomposition of pollutants in the soil [28]. The in situ bioremediation by the degrading bacterium DC-1 promoted the release of soil nutrients and increased their bioavailability [61]. In this investigation, the presence of strain DC-1 accelerated the restoration of biotic enzyme activities in butachlor-contaminated soil, which could rapidly remediate butachlor-contaminated soil, favoring crop growth and reducing soil stress.

### 3.7. Impact on Soil Microbial Community

Butachlor residue in the soil can influence the structure and vitality of the soil microbial population, hence damaging soil structure and fertility; as a result, the soil microbial community and structure have changed, impacting the soil’s ecological environment. Therefore, the repair of the microbial community structure in soil polluted with butachlor is of special significance. In this investigation, the high-throughput sequencing of 16S rRNA was used to identify bacterial genera and we investigated the α-diversity and β-diversity of soil microbial communities and discovered that the abundance and diversity of soil microbial communities altered following the application of butachlor, which corresponded with the prior research [53].

**Figure 5:**
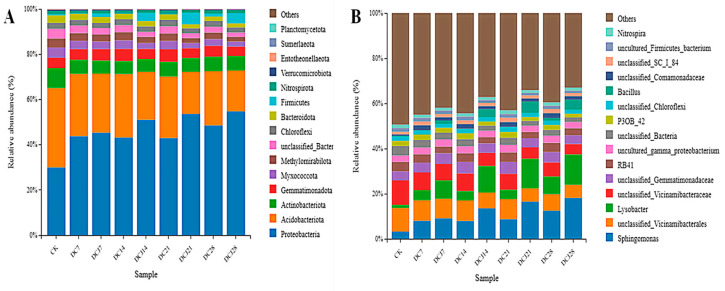
Relative abundance of soil bacterial community at phylum level (**A**) and genus level (**B**). **Note:** DC7, DCJ7, DC14, DCJ14, DC28, and DCJ28 represent day 7, day 14, and day 28, respectively.

At the phylum level, following the application of butachlor in soil, there was a decrease in the abundance of *Acidobacteriota*, *Actinobacteriota*, *Myxococcota*, *Methylomirabilota*, *Bacteroidota*, and *Firmicutes*, while *Proteobacteria* exhibited a noticeable increase (Figure 5A). The subsequent inoculation with strain DC-1 resulted in significant increases in *Actinobacteriota*, *Proteobacteria*, and *Firmicutes*, alongside the decreased abundance of *Acidobacteriota* and *Myxococcota*. *Firmicutes* primarily facilitated the degradation of butachlor, thereby accelerating its breakdown in the soil. Thus, strain DC-1 had an impact on the relative abundance of particular bacteria in the soil, guiding the microbial community in a more stable and beneficial direction. At later stages of the culture, the abundance of each strain stabilized by *Proteobacteria* (42.96–54.77%), *Acidobacteriota* (18.03–27.20%), *Actinobacteriota* (6.11–6.42%), *Gemmatimonadota* (4.15–5.54%), and *Firmicutes* (1.22–4.47%). Studies have indicated that *Proteobacteria*, *Actinobacteriota*, *Bacteroidetes*, and *Firmicutes* are beneficial bacteria for crops [62]. *Actinobacteria* play ecological roles in ecosystems, exhibiting bioremediation capabilities for organic and inorganic pollutants [60]. *Gemmatimonadota*, found in soil, performs certain environmental roles and is resistant to environmental stress [63]. The higher abundance of *Gemmatimonadota* in the DC compared to the DCJ and CK suggests that strain DC-1 mitigates environmental stress, thereby enhancing soil ecological conditions. Wu discovered both native microorganisms and foreign bacteria had synergistic effects [36], which affected the makeup of soil microbial communities and encouraged the proliferation of petroleum-degrading bacteria in soil [32]. In this investigation, in the butachlor-contaminated soil, strain DC-1 changed the structural makeup of the microbial communities. Additionally, it was able to work in concert with the native microorganisms to encourage the establishment of degrading bacteria, which was advantageous for the rehabilitation of the contaminated soil.

At the genus level, significant shifts in bacterial abundance were observed during the cultivation process. In the DC that received only butachlor, the abundance of *Sphingomonas* and *Lysobacter* steadily rose, but the abundance of *unclassified_Vicinamibacterales* and *unclassified_Vicinamibacteraceae* declined with time. These changes were more pronounced in the DCJ, indicating that strain DC-1 facilitated these shifts (Figure 5B). The abundance of inoculated *Bacillus* also underwent noticeable changes. In the CK, *Bacillus* accounted for only 0.44% of the entire bacterial microbiome. After a period of cultivation, *Bacillus* comprised 5.07% and 4.47% in DCJ21 and DCJ28, respectively, while in DC21 and DC28, these percentages were 0.063% and 1.2%. *Bacillus sphaericus* is an agriculturally important soil bacterium that can degrade herbicides into non-toxic forms [64]. In the later stages of cultivation, after butachlor degradation was complete, *Bacillus* maintained a certain level of stability in abundance, suggesting that *Bacillus* strains can survive on other substances in the soil after utilizing butachlor, thereby maintaining microbial community equilibrium. The inoculation with exogenous strains has been proven to modify the soil microbial population, which in turn changes the soil’s physical qualities [35]. Some studies have shown that the addition of *Bacillus subtilis* is beneficial to the remediation of Cd-As-Ni co-contaminated soil, can also reduce the abundance of *Fusarium oxysporum*, and reduce the incidence rate of plant wilt. Increasing beneficial bacterial communities can improve polluted soil and promote plant growth [65,66]. In this investigation, strain DC-1 increased the structural diversity of the soil microbial community and hastened the breakdown of butachlor. It has a positive effect on soil biodiversity and ecosystem stability.

The findings presented above have been established according to the findings of high-throughput sequencing. Studies have shown that after inoculation with strain DC-1, the microbial abundance in the soil changes, and the abundance of beneficial bacteria increases and maintains a certain equilibrium. Wu discovered that foreign bacteria and native microorganisms had a synergistic effect, promoted the colonization of soil by petroleum-degrading bacteria, and the composition of the soil microbial community was affected [32,36]. Furthermore, degrading bacteria can modify the structure of the soil’s microbe population and accelerate the decomposition of pollutants in contaminated soil [28]. In this investigation, strain DC-1 increased the quantity of microorganisms in butachlor-contaminated soil and might collaborate with native microorganisms to boost the emergence of degrading bacteria, increasing butachlor breakdown in the soil and enabling the remediation of the contaminated soil.

The redundancy analysis was used to investigate the association between strain DC-1 and the dominant strain in the soil, soil enzyme activity, and butachlor residue levels (Figure S3). Axis 1 explained 54.18% of the variance and Axis 2 explained 8.33% of the variance for a total explanation of 62.51%. Strain DC-1 had positive associations with *Lysobacter* and *Sphingomonas*, but negative relationships with *unclassified_Vicinamibacterales*, *unclassified_Vicinamibacteraceae*, and *RB41*. Strain DC-1 had a negative correlation with butachlor residue, a positive correlation with catalase, cellulase, and sucrase, and a negative correlation with urease. Butachlor residue correlated positively with urease and negatively with the other three enzymes. Catalase, cellulase, and sucrase showed positive correlations with *Lysobacter* and *Sphingomonas*, and negative correlations with the other three bacterial genera, contrasting with the relationship of urease with dominant bacterial genera in soil. Research has shown that there is a certain correlation between *Paenarthrobacter ureafaciens* ZF1 and soil enzymes and bacterial communities, and soil enzymes can also affect species and bacterial populations [67]. The interplay between soil microorganisms and enzymes contributes jointly to maintaining soil microbial stability.

## 4. Comment

In this investigation, we screened and identified a butachlor-degrading strain DC-1, *Bacillus cereus*, for which the optimal incubation conditions were 32.89 °C, pH = 7.29, and inoculum amount of 5.18%, and the degradation rate reached 87.06%. It was hypothesized that the degradation pathway of butachlor by strain DC-1 might be as follows: initially, butachlor is metabolized by deoxygenase to 2-chloro-N-(2,6-diethylphenyl)-N-methylacetamide. Following that, 2-chloro-N-(2,6-diethylphenyl)acetamide is generated by N-demethylation, which is ultimately converted to 2,6-diethylaniline. In addition, in the bioremediation experiment of butachlor-ammonium-contaminated soil, strain DC-1 could remove 99.23% of butachlor-ammonium (100 mg·kg^−1^) from the soil within 12 d. Moreover, inoculation with DC-1 strain increases sucrase, cellulase, and urease activities in soil, and can maintain or even activate the activity of peroxidase; and strain DC-1 affects the abundance and structure of certain species in soil microbial communities, accelerates microbial community succession, and promotes community evenness and diversity.

## Figures and Tables

**Table 1:** Physiological and biochemical characteristics of strain DC-1 *.

Detection Metrics	Detection Results	Detection Metrics	Detection Results
Glucose	+	Citrate salt	-
Non-Xylose	-	VP Response	+
Non-maltose	+	Nitrate reduction	+
Amylum	+	Gelatin liquefaction	+
Urea	-	Lysine decarboxylaselysine decarBoxylase	-
Hydrogen sulFide	-	Ornithine decarboxylase	-
Hesperidin	+	Arginine dihydrolase	-

* “+” indicates positive; “-” indicates negative.

## Data Availability

The original contributions presented in the study are included in the article/Supplementary Material, further inquiries can be directed to the corresponding authors.

## Supplementary Materials

The following supporting information can be downloaded at https://www.mdpi.com/article/10.3390/microorganisms12122568/s1, Table S1: Box-Benhnken test design and results of strain DC-1, Table S2: Analysis of variance for a response polygon regression model, Figure S1: Response plot of three interaction factors to the degradation of butachlor. Interaction of temperature and Ph (A). Interaction of temperature with inoculum size (B). Interaction of pH with inoculum volume (C). The degradation curve of the strain DC-1 before and after optimization (D), Figure S2: Residues of butachlor in soil with treatments A–D during the incubation period. Figure S3: Redundant analysis of relationships between soil enzymes, bacterial bacteria, strain DC-1, and butachlor. *p*-value: *p* = 0.001; and, *p* < 0.05.
