# Supplementary material for: The Degradation Characteristics and Soil Remediation Capabilities of the Butachlor-Degrading Strain DC-1
Source: Microorganisms. 2024 Dec 13;12(12):2568. doi: 10.3390/microorganisms12122568 (PMC11677828; doi:10.3390/microorganisms12122568)
Supplement: Supplementary file 1 [file microorganisms-12-02568-s001.zip › microorganisms-3298249-supplementary.pdf]

Table S1. Box-Benhnken test design and results of strain DC-1

Number	Temperature（℃）	pH value	Inoculation quantity	Degradation rate（%）
1	1	1	0	41.3
2	0	0	0	81.6
3	0	0	0	78.4
4	0	1	1	28.2
5	-1	1	0	17.3
6	-1	0	-1	14.5
7	1	-1	0	31.2
8	0	1	-1	33.7
9	0	0	0	82.9
10	0	-1	-1	14.3
11	0	-1	1	19.9
12	1	0	1	42.3
13	-1	-1	0	12.7
14	0	0	0	77.6
15	1	0	-1	27.9
16	0	0	0	80.8
17	-1	0	1	18.4

Table S2. Analysis of variance for a response polygon regression model

Source	Sum of squares	Freedom	Mean square	F value	F value
model	11858.96	9	1317.66	107.88	< 0.0001
temperature（A）	796.01	1	796.01	65.17	< 0.0001
pH（B）	224.72	1	224.72	18.4	0.0036
inoculation amount（C）	42.32	1	42.32	3.46	0.0051
AB	7.56	1	7.56	0.62	0.4572
AC	27.56	1	27.56	2.26	0.1768
BC	30.8	1	30.8	2.52	0.1563
A2	2944.02	1	2944.02	241.03	< 0.0001
B2	3346.6	1	3346.6	273.98	< 0.0001
C2	3311.08	1	3311.08	271.08	< 0.0001
total residual	85.5	7	12.21		
mismatch error	65.91	3	21.97	4.49	0.0906
pure error	19.59	4	4.9		
sum	11944.46	16			

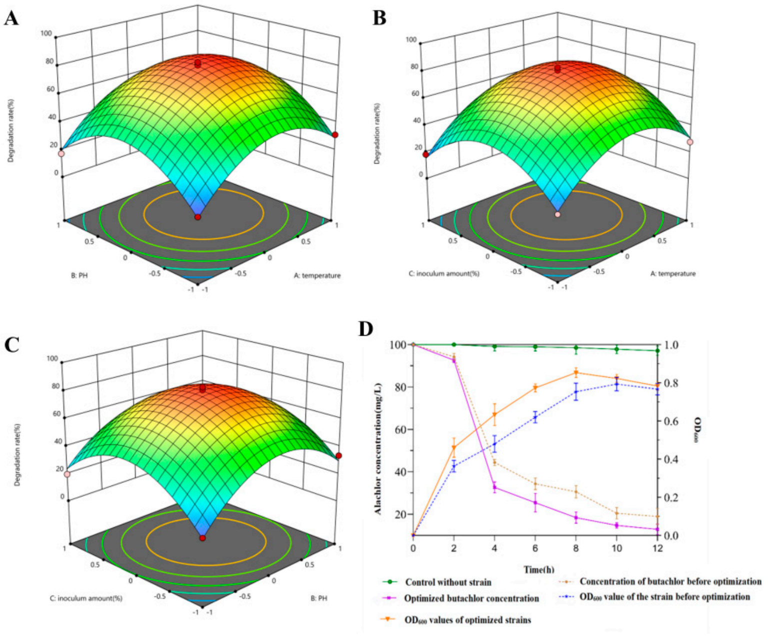

Figure S1. Response plot of three interaction factors to the degradation of butachlor. Interaction of temperature and Ph(A). Interaction of temperature with inoculum size(B). Interaction of pH with inoculum volume(C).The degradation curve of the strain DC-1 before and after optimization(D)

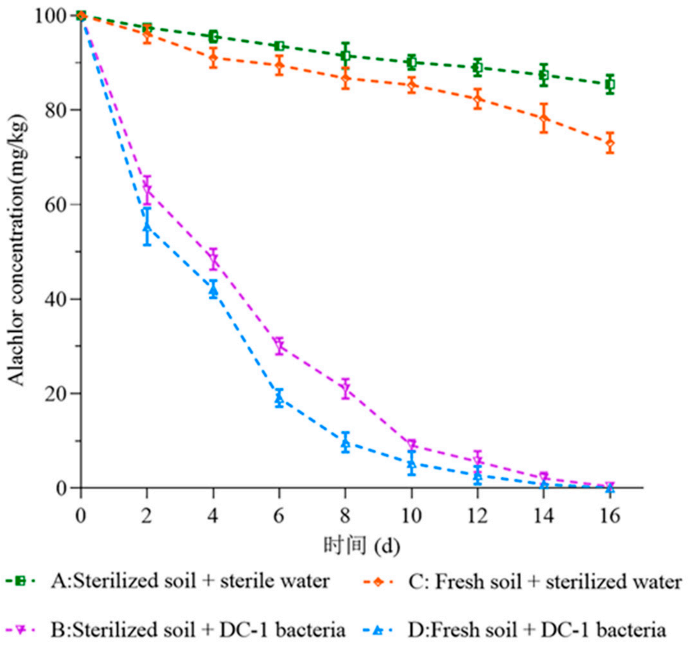

Figure S2. Residues of butachlor in soil with treatments A-D during the incubation period

Note: A: sterilized soil (250 g) + sterile water (8 mL), B: sterilized soil + DC-1 bacterial solution (8 mL, 1× 10^8 cfu • L^{-1}),C: fresh soil (250 g) + sterile water (8 mL), D: fresh soil + DC-1 bacterial solution (8 mL, 1× 10^8 cfu • L^{-1})

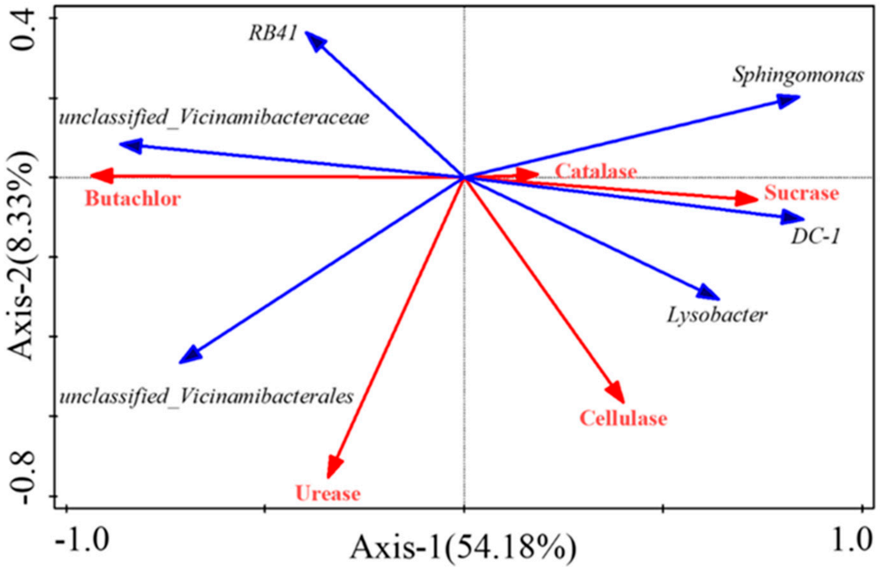

Figure S3. Redundant analysis of relationships between soil enzymes, bacterial bacteria, strain DC-1, and butachlor .P-value: *P*=0.001; and , P < 0.05.
